# The impact of severe osteogenesis imperfecta on the lives of young patients and their parents – a qualitative analysis

**DOI:** 10.1186/1471-2431-13-153

**Published:** 2013-09-30

**Authors:** Maman Joyce Dogba, Christophe Bedos, Michaela Durigova, Kathleen Montpetit, Trudy Wong, Francis H Glorieux, Frank Rauch

**Affiliations:** 1 Shriners Hospital for Children, 1529 Cedar Avenue, Montreal, QC H3G 1A6, Canada; 2 Faculty of Dentistry, McGill University, 3550 University Street, Montreal, QC H3A 2A7, Canada; 3 Department of Social and Preventive Medicine, Faculty of Medicine, Université de Montréal, C.P. 6128, Succ. Centre-Ville, Montreal, QC H3C 3J7, Canada

**Keywords:** Osteogenesis imperfecta, Qualitative study, Interpretative description, Parents, Canada

## Abstract

**Background:**

Osteogenesis imperfecta (OI) is a rare genetic disorder that causes increased bone fragility. Living with, caring for, and parenting a child with OI are all highly demanding and challenging. This study is a temporal analysis of the impact of severe OI on the lives of young patients and their parents.

**Methods:**

This study was carried out at the Shriners Hospital for Children, a pediatric orthopedic hospital located in Montreal, Canada. Using qualitative interpretative description, we conducted semi-structured interviews with 24 subjects – 12 young patients diagnosed with severe OI and 12 of their parents. The interview data were subject to a predominantly inductive open thematic analysis and a temporal comparative analysis. We did a retrospective chart review to complement our data collection.

**Results:**

We found that the impact of severe OI on the young patients and their parents was characterized by four themes: 1) Starting at the time of diagnosis, a series of stages shaped life and the return to every day “normal”, 2) Living with OI was full of “ups and downs” throughout life, 3) Every day “normal” life with OI consisted of significant changes for parents and challenges for the whole family, and 4) Living with OI generated some positive experiences.

**Conclusion:**

This study contributes to a better theoretical understanding of the impact of severe OI on families. It also has some practical implications for the development of effective support systems for patients with severe OI and their families.

## Background

Living with, caring for, and parenting a child with a chronic childhood disease are all highly demanding and can generate multiple challenges for the entire family. These challenges are not only health-related and medical in nature, but also emotional, psychosocial, financial, and educational [1,2]. In some cases, psychosocial support and financial aid are available, including programs like respite care, which help alleviate the burden of care for families [3]. However, support systems are often inadequate, and this is particularly true for rare genetic disorders [4].

Osteogenesis imperfecta (OI) is a rare genetic disorder that in the large majority of cases is caused by mutations in genes that code for collagen type I (the most common protein in bones, teeth, and skin). The main clinical characteristic is increased bone fragility, which varies widely in severity, ranging from intrauterine fractures and perinatal death to mild forms that remain asymptomatic until late in adult life [5,6]. Patients with severe OI surviving the neonatal period typically have short stature, limb and spine deformities and consequently restricted mobility. Such patients are usually diagnosed with OI types III or IV, according to the clinical Sillence classification [6,7].

Although a variety of therapeutic modalities are used to treat severely affected patients, they can, at best, mitigate the effects of the disease, but not cure it [8]. Orthopedic treatment with intramedullary rods is routinely used to straighten tibias and femurs and thus allow for ambulation. Rehabilitation through physiotherapy and occupational therapy promotes gross motor development and functional independence. Medical treatments like the intravenous infusion of bisphosphonates are used to support the other treatment modalities and reduce fracture rates and alleviate pain in the more severe forms of OI [6,9,10].

OI affects patients and families in many ways. Some studies have reported substantial negative consequences, such as persistent physical and functional limitations in adult patients, feelings of anger and guilt in parents, and social isolation in parents and patients [11-16]. By contrast, other studies have reported some positive effects and achievements in people with OI, such as a low incidence of depression, strong resilience, high educational level, and high employment rate [13,16-20].

Despite these prior studies, there is a dearth of information on how adolescents with severe OI and their parents experience day-to-day life. An understanding of these issues is crucial to design effective support systems. In this qualitative study, we therefore examined how adolescents and their parents experience living with severe OI.

## Methods

### Participants and setting

This study was carried out at the Shriners Hospitals for Children in Montreal, Canada, a specialized pediatric orthopedic hospital. At the time of the study (May 2011 to May 2012), this institution was actively following about 380 individuals with OI, aged 0 to 21 years, and residing mostly in Canada, the United States, and Latin America. The cost of care is covered by the organization; there are no out-of-pocket expenses for the patients or their parents. For many patients, the organization also assumes the costs for transportation, lodging, and meals [21].

The study included patients with a diagnosis of severe OI aged 15 to 21 years and their parents. All patients were receiving or had received treatment with intravenous bisphosphonates. The minimum age of study participants was selected to ensure sufficient maturity and the capacity to participate in interviews. Potential study participants were selected with the aim to capture a wide variation of life experiences (maximum variation) [22] and accordingly are from a variety of backgrounds and origins. For a patient to participate, at least one parent or legal guardian also had to agree to participate in an interview. Both patients and participating parents had to be fluent in either French or English to be eligible. We recruited twelve patients (9 female, 3 male) and 12 parents (8 mothers, 4 fathers). None of the parents was affected by OI.

The study was approved by the Institutional Review Board of McGill University. Patients 18 years and older signed a consent form, whereas those less than 18 years old signed an assent form. The parents also signed a consent form for their own participation and, if their child was under 18 years of age, a parental consent form.

### Research design

We opted for interpretative description [23] which examines a “clinical phenomenon with the goal of identifying themes and patterns among subjective perspectives, while accounting for variations between individuals” [24], p. 1285). The initial study phases were oriented toward identifying the professional, familial, social, and psychological impacts of severe OI. As patterns within the data became more apparent, we adopted a life course approach to study how temporal factors affected the lives of young patients and their parents.

The research team included two senior physicians responsible for the follow-up of patients with severe OI (F.R. and F.H.G.); a senior qualitative researcher in social and preventive medicine and social dentistry (C.B.); a postdoctoral fellow with a medical background plus training in qualitative health service research, but who was not part of the medical team (M.J.D.); a senior occupational therapist involved in previous research on patients with OI (K.M.); a social worker who supports patients with OI (T.W.); and a senior research assistant involved in research on patients with OI (M.D.).

### Data collection and analysis

The first author conducted the one-on-one semi-structured interviews with the patients and their parents during regular follow-up visits at the hospital. Patients still receiving bisphosphonate treatment were interviewed only after the infusion planned for the visit had been completed. Parents and patients could choose whether they preferred to be interviewed in the presence or absence of their child or parent, respectively. When there were two parents, they decided which one of them would be interviewed. The interviews took place in a quiet room close to the clinic area. Eight interviews were performed in French, the rest were held in English.

The interviewer used two different interview grids, one for patients and one for their parents. Interviews lasted approximately 45 minutes and were recorded using a tape recorder. After each interview, details about the interview (setting, participant’s reactions, etc.) were documented in written notes. Interviews were transcribed, anonymized and then analyzed using NVivo 8 qualitative analysis software (QSR International). Interviews were coded in the original interview language, but codes and themes were labelled in English. The authors translated illustrative quotations from French to English.

Clinical indicators of disease severity were collected through retrospective chart review. Surgical history was highlighted by the number of lower extremity segments (upper and lower leg on each lower extremity) that had undergone rodding surgery.

## Results

The study included 12 young patients with severe OI (3 male, 9 female) between 16 and 21 years of age and 12 parents. The diagnostic distribution of the patients was as follows: OI type III, N = 4; OI type IV, N = 5, OI type V, N = 3. Seven patients were from Canada, 3 from the United States and 2 from Europe. Six patients were unable to walk, one patient walked only at home, and 5 patients were able to walk in the community without assistance. They had suffered between 14 and 46 (mean: 25) long-bone fractures during their lifetime. The height score ranged from -8.0 to -1.0 (mean: -4.6). They had been followed at the Shriners Hospital for 12 to 17 years (mean: 14.5 years).

To provide an interpretative overview, the diagnosis of severe OI drastically changed the parents’ lives and sometimes was compared to an earthquake in that it was sudden and unpredicted. Then, after a period of acceptance, the parents moved into what could be called a “normal life with OI” characterized by ups and downs, changes, and some positive experiences.

Unlike parents, patients did not experience such an “earthquake”, as all of them were newborns or toddlers at the time of the diagnosis. Moreover, patients did not refer explicitly to any period of adaptation following the diagnosis. Rather than talking about the changes to their lives, patients described their “normal life with OI” as made up of ups and downs, challenges, and some positive experiences.

**Figure 1:**
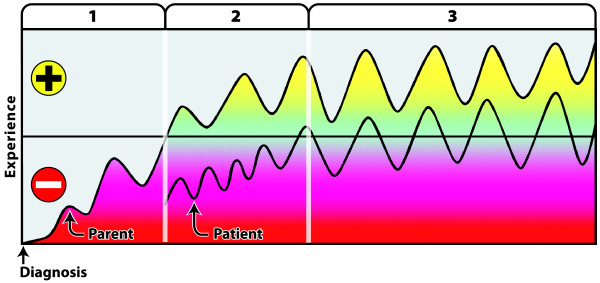
The impact of severe OI on parents and young patients.

A schematic temporal overview of living with severe OI during the first two decades of life is shown in Figure 1. The top curve shows the experience of parents, the bottom that of patients. The life cycle is divided up into three stages for parents, and two for patients. The ups and downs throughout life are represented by an oscillating black curve. The more difficult changes and challenges faced by the parents and patients are shown in red. Experiences considered positive are shown in green and yellow.

The thematic analysis revealed four distinct themes, as summarized in Table 1 and described below with illustrative quotes.

**Table 1:** Themes and subthemes from the thematic analysis of the impact of severe OI on young patients and their parents

**Themes**	**Subthemes**
1) Starting at the time of diagnosis, a series of stages shaped life and the return to every day “normal”	Stage 1: Parents experience the diagnosis as an earthquake
	Stage 2: Parents come to an acceptance of the diagnosis
	Stage 3: Parents gradually redefine a “new normal” life with OI
2) Living with OI was full of “ups and downs” throughout life	Times of crisis alternated with more calm stable moments
	Parents and patients alternated between negative and positive feelings
3) Every day “normal” life with OI consisted of significant changes for parents and challenges for the whole family	Changes guided by a fear of fracture and a concern about safety
	Changes related to the increased burden of care
	Patients tended to talk about challenges rather than changes
	Changes and challenges affected the whole-family, including siblings and the extended family
4) Living with OI generated some positive experiences	

### Theme 1: Starting at the time of diagnosis, a series of stages shaped life and the return to every day “normal”

We identified three main stages in the lives of parents following the diagnosis of OI: stage 1 – parents experience the diagnosis as an earthquake; stage 2 – parents come to an acceptance of the diagnosis; and stage 3 – parents gradually redefine a “new normal” life with OI. In contrast to the parents, patients only vaguely recalled the moment when they realized that they were different from other children, but having no prior reference to what life was like without OI, they referred to their life as “normal”: “I haven’t lived without it [OI] before, so I wouldn’t know how it is different”. Consequently the three stages were documented in parents only.

The three stages were not of equal length, and moving through them was not a linear process. Indeed, some parents quickly moved from stage 1 to stage 3, while others needed more time to accept the diagnosis. Moreover, the data suggest that the patients experienced the adaptation stage when their parents were redefining their “new normal life”.

#### Stage 1: Parents experience the diagnosis as an earthquake

In most cases, the process that led to the diagnosis of OI was sudden and unexpected. All parents used words like “devastation”, “shock”, and “loss” to describe their feelings. Most common were feelings like being “overwhelmed with sadness” or “depressed”; some expressed feelings of anger or guilt. The feelings of devastation were stronger in some parents who were not given much information at the time of the diagnosis.

“I was just in shock and there was no explanation as to what OI was, when I asked… I tried to ask question (…), they told me to go to library and research it; they don’t have time for this. I actually went out and bought a computer so that I could do more reading.”

In a few cases, the diagnosis also brought a sense of relief because it reduced the level of uncertainty and ruled out lethal conditions. For some parents, it also helped to dismiss suspicions of child abuse.

“I could have been more concerned… I mean, OI is not the worst on the list. But we were seriously impacted because it is … a big diagnosis …and because at the time, there was no treatment. And it still has no cure. I don’t want to say I was devastated because it is not, in her case, a lethal diagnosis, but I mean, it … well it took a huge emotional toll.”

#### Stage 2: Parents come to an acceptance of the diagnosis

Immediately after experiencing the “earthquake”, parents said they actively sought out information by reading and asking questions. They recounted that they had no choice but to move forward and help their child through life.

“It was very upsetting and discouraging but there was nothing we could do about it… Whatever we had to do to help her and keep her safe, we just accepted and did it.”

At this stage, as they sought out other families living with OI, many parents realized for the first time that there was no cure for OI. They eventually accepted that OI would be part of their life forever.

“Yeah, it was hard to hear initially, and we prayed so that he survived. It was hard because they said that there is no cure. After that, within about a month actually, things returned to normal, and we had to face it.”

#### Stage 3: Parents gradually redefine a “new normal” life with OI

Once parents realized that they had to live with OI, they moved to what can be called the “new normal” life. At this stage, the parents and patients said they tried to live as normally as everybody else, despite the increased challenges in all aspects of their lives. The parents said they understood that life would never be how they had envisioned it would be.

“We’ve tried to maintain as much of a normal life as possible. But that is also very tiring, always trying to maintain normality when things aren’t normal. It takes a lot of energy from everyone involved.”

### Theme 2: Living with OI was full of “ups and downs” throughout life

Both parents and patients described the impact of OI in terms of “continuous ups and downs”. On the one hand, times of crisis alternated with more calm stable moments; on the other hand, parents and patients alternated between negative and positive feelings.

#### Times of crisis alternated with more calm stable moments

For parents, repetitive fractures constituted one of the most difficult aspects.

“It goes up and down. It depends if you have a year where there is break after break after break. This type of year is very hard. Then you hit a stage where there are not so many breaks, so things get easier. The challenges just come and go. They are some years that are very difficult, and other years that are easier.”

The patients also stressed that fractures were particularly difficult to cope with.

“I mean, today might be a bad day, where you break, but then tomorrow could be way better and something good could come out of that.”

#### Parents and patients alternated between negative and positive feelings

While difficult events were usually associated with sadness and negative feelings, stable moments brought hope. However positive and negative feelings could occur simultaneously *(concurrent contradictory feelings)*, or follow each other *(successive contradictory feelings)*. In fact, concurrent contradictory feelings were reported particularly at the time of birth, where the excitement of being a new parent coexisted with sadness, anxiety, and uncertainty about the future. One parent said: “It was, on one hand, exciting that she was born; on the other hand, being told that something could potentially be wrong.”

### Theme 3: Every day “normal” life with OI consisted of significant changes for parents and challenges for the whole family

The parents reported a lot of changes in their lives following the diagnosis of OI. These changes could be classified as temporary or definite. At times, they were driven by a fear of fracture and a concern for safety; at other times, they were the consequence of the increased burden of care. The changes could start immediately after the diagnosis or arise gradually as needs changed and means permitted, and they had repercussions in a number of different spheres, including professional, marital, and social.

#### Changes guided by a fear of fracture and a concern for safety

Parents reported that living with OI on a daily basis meant living with many fears and sources of stress. The constant fear of fracture and the resulting concern for safety led to changes in daily activities and oriented major decisions in life. For example, the concern for safety affected planning for holidays, the spatial layout of the home, choosing a school, and participating in social activities. Parents restricted outdoor activities, especially when children were younger and when other children were around.

The parents reported selecting schools equipped with functional elevators and handicap-accessible transportation systems, and also looking for teachers who were comfortable with having a child with OI in their class, which was not always the case.

Furthermore, whether homeowners or tenants, the parents gradually changed their homes to suit their child with OI. For example, they lowered light switches and bathroom sinks, made their home wheelchair accessible, and, whenever possible, lived in a one-level house with no stairs.

#### Changes related to the increased burden of care

The increased burden of caring for a child with OI sometimes impacted the parents’ professional lives and finances. For many parents, mostly mothers, the diagnosis of OI resulted in a dilemma: continue their professional activities, or quit and stay home. Dealing with disability on a daily basis, managing a large number of medical appointments and a lack of safe daycare proved incompatible with full-time employment. Many parents stopped their professional activities when the children were very young. As one parent reported: “I left my position as a community health nurse because I realized that I couldn’t just send a child in a body cast to my baby sitter while I went to my job.”

In some cases of a definitive interruption of professional activities, the parents had already decided on having a “stay-at-home parent” prior to the diagnosis. In other cases, the parent leaving their job was temporary: parents who were students or self-employed at the time of the diagnosis sometimes returned to part-time professional activities. Other parents managed to return to work but experienced reduced working hours or re-oriented their career: “It really affected my professional career … I spent a lot of time designing and building our new house. You know, it really changed the trajectory of my career”.

Many families reported a strain on the family’s finances as a result of these professional changes due to frequent medical visits and related treatment expenses. One participant stated: “So we were living with less money than we should have been… day-to-day life was missing work. It’s been very expensive”. Another participant said: “Even though the Shriners pay for surgeries and stuff, it costs to travel and means time off from work”.

#### Patients talked more about challenges than changes

The patients tended not to describe the impact of OI on their lives in terms of changes. Instead, they talked about challenges related to education and in accepting their disabilities at adolescence.

“… the first few years of high school, grade 8 to grade 10, were very hard for me, because at the school that I was going to at the time, a lot of teenagers … looked at the outside first, before looking at the inside, at your personality, and I found that really hard.”

The patients experienced other challenges as well.

“Not being able to walk and stuff like that, I mean I always try to keep moving forward, keep standing and walking, and… I try to keep getting stronger and stuff. My fear is probably taking one step forward and then two steps back, like when you break.”

“I think, for me, it is just the little things and not so much the big things. For instance, reaching something or relying on other people to do certain things for you. Everything for me, like personal care, or being in the kitchen, is pretty hard. Nothing is really adapted to my needs. Just, you know, getting in the car or little things like that, I find are daily challenges …”

### Changes and challenges affected the whole family, including siblings and the extended family

According to both parents and patients, the huge amount of attention required by the child with OI affected siblings and even the extended family. On example was the impact on family activities.

“We didn’t want to be separated as a family so we changed many of the things we did together. For instance, we couldn’t ski anymore. We had always skied as a family, but it wasn’t until the last few years, that my children, now teenagers, went skiing; because we didn’t want to go and leave her at home, and think about her being sad because she was missing out.”

“I know, my sisters also feel sad for me. But we don’t talk a lot about OI. They cannot do what they want when I am sick. Yeah”

It also affected family dynamics. Parents said they usually gave the child with OI increased attention, which meant less time and energy for siblings and their spouse. Consequently sometimes siblings felt abandoned and relationships between parents suffered.

“The siblings suffer quite a bit. They suffer because they are always second; there is no way that you can make them as important when you have a child that has severe disabilities.”

“Elsie’s^a^ father and I divorced while she was quite young because it was very taxing on our relationship.”

### Theme 4: Living with OI generated some positive experiences

While the disease itself was never considered a positive event, it did generate some experiences that were considered beneficial for parents and patients. These included very positive experiences for parents and patients with specialized care centers and patients’ associations. The parents reported that their children came to look forward to their next hospital visit, to “getting time off school to go to hospital.” This was because these visits gave them the opportunity to meet families with similar experiences, as well as knowledgeable staff and understanding nurses.

“Well, going to the (…) hospital, a lot of times, was a positive experience, and also going … to the OI foundation, meeting all my friends there and just interacting with other people who have OI.”

Other positive experiences included the consolidation of relationships, and siblings becoming more sensitive and compassionate. Living with a child with OI drew some spouses closer together as they learned to share caring for their OI child and the stress involved.

A beneficial effect experienced by parents and patients was adopting a more positive mindset. In the face of the enormous challenges, many adopted a new way of thinking, one characterized by “putting things into perspective” and “living one day at a time”. They said they lived expecting that tomorrow would be better.

“We tried to enjoy things just in a very simple way… enjoy simple things in life because hard times come but things are going to get better and then it is going to be hard times again.”

“You have to live day to day, never take anything for granted… When I see somebody who had a minor break and will be down for a month … really this is nothing, I mean really nothing.”

Finally, some of the parents experienced some unexpected outcomes that came to be valuable and emotionally rewarding accomplishments. For example, some mothers became referral persons for doctors needing more information about OI, others visited patients with OI and shared their experiences with other parents, and others became spokespersons for disabled people.

## Discussion

Our temporal analysis of the impact of severe OI on patients and their parents shows that most parents experienced a great shock at the moment of diagnosis. They then gradually adapted to a “new normal” life characterized by ups and downs along with professional, marital and social changes. These changes affected the whole family, particularly siblings. The patients did not experience an initial shock because they had been diagnosed with OI as newborns or toddlers. However, they realized that they were different from other children and started adapting to a life characterized by ups and downs and increased challenges. Over time, the parents and patients developed a positive mindset and started considering some experience arising from OI as beneficial.

This temporal approach enhances our understanding of the impact of severe OI on parents and patients. Rather than being a frozen image of positive versus negative impacts, this study portrays the impact of severe OI as a process that changes over time. Such a conceptualization and the new insights it provides makes a valuable theoretical contribution by orienting future research on OI and other chronic genetic diseases [25].

This study also has several practical implications with respect to improving support services for people living with OI. A previous study called for a shift in the perspective of social services from a time-limited approach to a life-span approach in order to effectively handle the chronicity for OI and rare genetic diseases [26]. The findings of our study go further, suggesting that we enlarge the life-span approach to make it more family centered. Our study shows that parents and patients differ in their support needs and that needs of both vary over time. For parents, the period of diagnosis is particularly difficult, especially when it coincides with birth, an intense moment in the life cycle. Therefore, adequate support system focused on how the diagnosis is announced to more complex psychological counselling would be helpful [3]. After the period of the diagnosis, parents may still need support as they move into their “normal” life with OI. At this time, flexibility in the workplace and understanding from school teachers is crucial. Government financial assistance would help offset some of the financial burden placed on these families. In the same vein, respite care that includes the care of siblings would be helpful in alleviating the burden of care [3].

For patients, perhaps the most positive change would be having support services, psychologists, and schools working together to ensure the acceptance of disability. And because new multidisciplinary specialized centers that provide a life-span and family-centered support system are valuable for patients and parents, most of these centers – already working with social workers and/or psychologists [27] – should also work in collaboration with schools and workplaces.

Our study points to a number of areas for future investigation. First, we need to better understand the support needs of patients with severe OI beyond adolescence. For example, what are the needs of patients (and their families) when transitioning from the pediatric setting to adult care institutions? We also need to extend our perspective even further to explore the impacts of severe OI across generations. We need to add genetic counsellors to the support team, so young adults with severe OI can be informed about their reproductive choices.

Despite these findings and practical implications, this study has some limitations. First, while our research design based on the purposeful selection of severely affected patients yielded valuable data, such an approach may limit the generalization of our conclusions to other patients. Yet theoretical transfer is feasible for the clinical context defined by the boundaries in our study. It is also true that because we used a qualitative approach, we have no quantitative measure of the financial loss or impaired quality of life associated with OI, information that could be used to advocate for better support systems on behalf of families living with OI. Second, the sample size could have limited an exhaustive exploration of the impact of severe OI. Although an in-depth qualitative analysis was performed, further investigations are needed to examine variations in this impact according to severity of the disease.

## Conclusions

In conclusion, living with a diagnosis of severe OI can be devastating for parents, yet most of them eventually redefine life, creating a “new normal” life characterized by ups and downs, challenges and change, but also positive experiences. Patients with severe OI also face many challenges, and they too tend to develop a positive mindset. These findings reveal a number of specific support needs and suggest that these needs would be best met by a tailored life-span and family-centered support system delivered through specialized genetic disease centers.

## Endnote

^a^Fictitious name.

## Competing interests

The authors declare that they have no competing interests.

## Authors’ contributions

MJD: designed the data collection instruments, gathered the data, carried out the initial analyses, drafted the initial manuscript and approved the final version as submitted. CB: conceptualized and designed the study, supervised the data analysis and interpretation, critically reviewed and revised the manuscript and approved the final version as submitted. MD: participated in data interpretation, reviewed and revised the manuscript and approved the final version as submitted. KM: participated in data interpretation, reviewed and revised the manuscript and approved the final version as submitted. TW: participated in data interpretation, reviewed and revised the manuscript and approved the final version as submitted. FHG: reviewed and revised the manuscript and approved the final version as submitted. FR: conceptualized and designed the study, participated in the interpretation of data, reviewed and revised the manuscript and approved the final version as submitted.

## Pre-publication history

The pre-publication history for this paper can be accessed here:

http://www.biomedcentral.com/1471-2431/13/153/prepub
